# Characteristics of enzymolysis of silkworm pupa protein after tri-frequency ultrasonic pretreatment: kinetics, thermodynamics, structure and antioxidant changes

**DOI:** 10.3389/fbioe.2023.1170676

**Published:** 2023-06-23

**Authors:** Shuangmei Ge, Chunyan He, Yichen Duan, Xiaotao Zhou, Jialong Lei, Xiangyun Tong, Libing Wang, Qiongying Wu, Junqiang Jia

**Affiliations:** ^1^ School of Biotechnology, Jiangsu University of Science and Technology, Zhenjiang, China; ^2^ School of Grain Science and Technology, Jiangsu University of Science and Technology, Zhenjiang, China

**Keywords:** protein, dynamics, thermodynamics, antioxidant, enzymolysis

## Abstract

As a by-product of the sericulture industry, the utilization rate of silkworm pupa resources is currently not high. Proteins are converted into bioactive peptides through enzymatic hydrolysis. Not only can it solve the utilization problem, but it also creates more valuable nutritional additives. Silkworm pupa protein (SPP) was pretreated with tri-frequency ultrasonic (22/28/40 kHz). Effects of ultrasonic pretreatment on enzymolysis kinetics, enzymolysis thermodynamics, hydrolysate structure as well as hydrolysate antioxidant of SPP were investigated. Ultrasonic pretreatment significantly increased the hydrolysis efficiency, showing a 6.369% decrease in *k*_m_ and a 16.746% increase in *k*_A_ after ultrasonic action (*p* < 0.05). The SPP enzymolysis reaction followed a second-order rate kinetics model. Evaluation of enzymolysis thermodynamics revealed that Ultrasonic pretreatment markedly enhanced the SPP enzymolysis, leading to a 21.943% decrease in *E*_a_. Besides, Ultrasonic pretreatment significantly increased SPP hydrolysate’s surface hydrophobicity, thermal stability, crystallinity, and antioxidant activities (DPPH radical scavenging activity, Fe^2+^ chelation ability, and reducing power). This study indicated that tri-frequency ultrasonic pretreatment could be an efficient approach to enhancing the enzymolysis and improving the functional properties of SPP. Therefore, tri-frequency ultrasound technology can be applied industrially to enhance enzyme reaction process.

## 1 Introduction

Silkworm pupa is a nutritious edible insect containing a rich source of micronutrients, such as iron, zinc, riboflavin, pantothenic acid, and folic acid (He W. et al., 2021), and very popularly consumed in the Asian region, including Korea and China, etc. (Li et al., 2019). The protein content of silkworm pupa is high (48%–60%), consisting of 18 amino acids, with 8 essential ones accounting for 42% of the total amino acid content (Zeng et al., 2021). Therefore, it is necessary to study the nutritional value of silkworm pupa protein (SPP) extracted as a by-product. Recently, enzymatic hydrolysis was used to improve the nutritional value of protein (Zhang et al., 2017; de Lima Alves et al., 2020). For example, Valdez-Ortiz et al. (2012) reported that the bean protein hydrolyzed-based products presented a good balance of essential and aromatic amino acids related to nutraceutical properties. In addition, Tironi and Añón, (2010) reported that the *amaranth* protein after hydrolysis exhibited strong antioxidant activity. SPP hydrolyzed peptides have been demonstrated to have blood pressure-lowering effects (Tao et al., 2017). In a similar report, the tripeptide obtained after isolation and purification of SPP by ultrasound treatment exhibited significant angiotensin-converting enzyme (ACE) inhibitory activity (Jia et al., 2015). In addition, SPP has also demonstrated anti-cancer properties (Ji et al., 2022). Therefore, the peptides derived from the enzyme hydrolysis of SPP possess high medicinal value.

Ultrasound has been widely used as a green and efficient physical treatment technique to assist enzyme hydrolysis (Gai et al., 2022; Hong et al., 2022). The cavitation and shearing effects of ultrasound have been shown to introduce changes in protein structure and molecular weight (Uluko et al., 2014), influencing the nature of the enzymatic products and enhancing the biological activity of the hydrolyzed peptides. Sonication increased the hydrolysis of *β*-lactoglobulin by pepsin and trypsin while improving its antioxidant activity and protein hydrolysis products (Ma et al., 2018). The improvement in hydrolyzed peptides was particularly evident with multi-frequency ultrasound, making it an effective pretreatment method to improve proteolysis. For example, casein hydrolysis was significantly enhanced after pretreatment with multi-frequency ultrasound (20/40/60 kHz). In a previous study, the influence of ultrasonic treatment with different frequencies (22, 28, and 40 kHz) on the extraction rate and physicochemical properties of SPP was investigated. Results showed that ultrasonic treatment with tri-frequency (22/28/40 kHz) significantly increased the protein extraction rate, modified protein structure as well as significantly improved protein solubility and antioxidant activity (Ge et al., 2022). However, the influence of ultrasonic treatment with tri-frequency on characteristics of SPP enzymolysis has not yet been reported.

In this work, the effect of tri-frequency ultrasound treatment on the kinetics and thermodynamics of enzymatic hydrolysis of SPP. Fluorescence spectroscopy, differential scanning calorimetry (DSC), and X-ray diffraction (XRD) were used to analyze the structure changes of SPP hydrolysate. The antioxidant activity of SPP hydrolysate was also evaluated by DPPH radical scavenging and Fe^2+^ chelation and reducing power.

## 2 Materials and methods

### 2.1 Materials

Silkworm pupa (China, 68% protein, Kjeldahl method) is crushed and passed through 100 mesh screens. Degrease the pupa completely with hexane, and dry the sample. Then stored in a refrigerator at 4°C for subsequent experiments. Alkaline protease (200 U/mg) is provided by Shanghai Yuanye Biotechnology Co., Ltd. Other reagents used in this study are analytical-grade chemical reagents.

#### 2.1.1 The treatment of SPP with tri-frequency ultrasound

The protocol of tri-frequency ultrasound treatment was selected from the optimal conditions, which were studied in our previous studies (Ge et al., 2022). Disperse silkworm pupa powder (10 g) in 500 mL of pure water. Then, adjust the pH to 9.0. The suspension was treated using a multi-frequency ultrasonic reaction generator (which was developed by Jiangsu University, water bath, Nanjing, China) at optimal conditions (ultrasonic temperature 50°C; ultrasonic power 50 W; ultrasonic frequency 22/28/40 kHz; ultrasonic time 60 min; pulse durations of on-time 1 s and off-time 1 s). After ultrasound, the treated suspension was centrifuged. Then its supernatant was collected and adjusted to pH 3.9 using 1 mol/L HCl to precipitate the protein (treated SPP). The precipitate (treated SPP) was washed several times with distilled water, freeze-dried, and subjected to further experiments.

### 2.2 Enzymatic hydrolysis

Different concentrations of SPP (2, 4, 6, and 8 g/L) were dissolved and dispersed with a pure water solution, they were preheated at different temperatures (20, 30, 40, and 50°C) for 20 min and then adjusted to different pH (Tironi and Añón, 2010; Jia et al., 2015; Tao et al., 2017) with 1 mol/L NaOH and HCl. Different concentrations (0.2, 0.4, 0.6, and 0.8 g/L) of alkaline protease were added to initiate an enzymatic process lasting about 90 min. Several drops of NaOH (0.1 mol/L) were added to maintain the optimal pH. For the study of enzymatic kinetics, the hydrolyzed (1 mL) samples were removed at 5 min intervals at the beginning of 25 min, after which the interval was increased to 10 min until the end of the 90-min enzymatic reaction period. The reaction was stopped by keeping the samples in boiling water for 10 min. It was further cooled to room temperature, pH adjusted to 7 (neutralized) with 1 mol/L NaOH and HCl, and then centrifuged (10,000 r/min, 10 min, 4°C). The recovered supernatant was freeze-dried and then stored at −4°C for structural studies.

### 2.3 Determination of hydrolysis degree

DH was determined using the method proposed by Adler-Nissen (Adler-Nissen, 1986). The DH of SPP was calculated according to Eq. (1).

$$DH\left(\%\right)=\frac{C\times V}{{\alpha \times M}_{p}\times h_{total}}\times 100$$ (1)

Where, *C*: concentration of NaOH (mol/L); *V*: volume of NaOH consumed (mL); α: degree of dissociation of the amino group (α: 0.44); *M*_p_: mass of the substrate protein (g); h_total_: total number of peptide bonds of the substrate protein (SPP of h_total_: 8.2 mmol/g).

### 2.4 Determination of the concentration of hydrolyzed SPP

The concentration of hydrolyzed SPP was determined according to Dabbour et al. (2021). Thus, the kinetic parameters of enzymatic protein hydrolysis were determined. The hydrolyzed pupa protein concentration of SPP was calculated according to Eq. (2).

$$C_{t}=DH\times C_{i}\times 0.01$$ (2)

Where, *C*_t_: hydrolyzed pupa protein concentration (g/L), *C*_i_: initial protein concentration (g/L). DH: the degree of hydrolysis.

### 2.5 Determination of kinetic parameters k_m_ and k_A_ and initial reaction rate

The initial reaction rate were measured by the method described by Jia et al. (2010). The Michaelis-Menten equation kinetic model was used to study the hydrolysis kinetics of SPP.

$$V=\frac{k_{A}E_{c}C}{k_{m}+C}$$ (3)

Where *V*: initial reaction velocity (g/L*min^-1^); *k*_A_: mean value of apparent decomposition rate constant or mean value of enzyme-substrate binding frequency (min^-1^); *E*_c_: enzyme concentration in the reaction system (g/L); *C*: total initial substrate peptide bond concentration. (g/L); *k*_m_: Mie’s constant (g/L). Eq. (3) was transformed into the following equation.

$$\frac{1}{V}=\frac{k_{m}}{k_{A}E_{c}}\times \frac{1}{C}+\frac{1}{k_{A}E_{c}}$$ (4)

The inverse of the initial velocity of the reaction (1/*V*) was plotted against the inverse of the initial peptide bond concentration of the substrate (1/*C*), and the values of *k*_m_/*k*_A_ *E*_c_ and 1/*k*_A_ *E*_c_ could be obtained from the linear regression equation, and then *k*_m_ and *k*_A_ were calculated.

### 2.6 Determination of enzymatic reaction kinetics (k)

To determine the reaction rate constant *k*, the enzyme hydrolysis reaction can be treated as a primary reaction with the reaction kinetic model as (Kadkhodaee and Povey, 2008).

$$\frac{{dC}_{t}}{dt}=-kC_{t}$$ (5)

Where, *C*_t_: concentration of SPP at reaction time (g/L); *t*: reaction time (min); *k*: reaction rate constant (min^−1^).

After integrating, the model can be expressed as follow.

$${In}_{C_{t}}=-kt+{In}_{C_{0}}$$ (6)

Where, *C*_0_: initial value of protein concentration (g/L), *t*: hydrolysis time (min), *k*: reaction rate constant, as it is difficult to measure the protein reduction, the reaction rate can be reflected in the increase of peptide content released from SPP. To some extent temperature and pressure, *C*_0_ = *Q*_∞_ and *C*_t_ = (*Q*_∞_—*Q*_t_). Thus, it can be written as Eq. (7).

$$In\left(Q_{\infty }-Q_{t}\right)=-kt+\ln Q_{\infty }$$ (7)

Where, *Q*_t_: peptide concentration at a given time *t* (g/L), *Q*_∞_: peptide concentration released from the ultimate sericin (g/L), then by the above equation as ln (*Q*_∞_-*Q*_*t*_) ∼ *t* plotted as a straight line, the slope of the line is the reaction rate constant k.

$$k=k_{c}+k_{u}$$ (8)

Where, *k*: represents the total ultrasonic reaction rate constant; *k*_c_: the reaction rate constant caused by temperature, min-1; *k*_u_: the reaction rate constant caused by ultrasound (min^-1^).

### 2.7 Thermodynamics of enzymatic protein hydrolysis

A 50 mL volume of traditional and treated SPP (2 g/L) was adjusted to pH 9.0 and subjected to enzyme hydrolysis sing alkaline protease (0.02 g/L) at temperatures of 20, 30, 40°C and 50°C, respectively. The temperature dependence of the rate constant kin can be described by the Arrhenius equation.

$$k=A^{\frac{-E_{a}}{RT}}$$ (9)

Where, *A*: the exponential factor or collision factor (min^−1^), *E*_a_: activation energy (J/mol), *R*: universal gas constant [8.314 J/(molk)], and *T*: the Kelvin temperature.

Solving Eq. (9) yields the logarithmic equation that:

$$lnk=lnA-\frac{E_{a}}{RT}$$ (10)

Eq. (10) is used to calculate *E*_a_ and A by plotting ln_*k*_ versus 1/*T*. The plot of ln_*k*_ versus 1/*T* should give a straight line. The slope and intercept are -*E*_a_/R and ln_*A*_, respectively, from which *E*_a_ and *A* can be calculated.

According to the Eyring transition state theory, the relationship between the activation entropy and activation enthalpy with the reaction temperature and the reaction rate constant can be obtained as.

$$\ln\left(\frac{k}{T}\right)=-\frac{\Delta H}{R}\cdot \frac{1}{T}+\frac{\Delta S}{R}-\ln\left(\frac{h}{k_{B}}\right)$$ (11)

where, *k*_B_: Boltzmann’s constant (1.38 × 10^-23^ J/K), *h*: Planck’s constant (6.6256 × 10^-34^ J s), *ΔG*: Gibbs free energy of activation (J/mol); *ΔH*: enthalpy of activation, J/mol; *ΔS*: entropy of activation, J/(molK).

Therefore, the enthalpy of activation and Gibbs free energy was calculated according to Eq. 12, 13.

$$E_{a}=∆H-RT$$ (12)

∆G=∆H−T∆S (13)

### 2.8 Second order rate extraction model

Second order rate model was determined using the method proposed by Agu et al. (2022). The formula was calculated according to Eq. 14.

$$\frac{t}{C_{t}}=\frac{1}{K{C_{s}}^{2}}+\frac{t}{C_{s}}$$ (14)

Where: *C*_t_: peptide yield at time *t*, *t*: enzymolysis time (min), *C*_S_: peptide yield at saturation (g/mL). *K*: second order extraction rate constant.

### 2.9 Surface hydrophobicity analysis

Surface hydrophobicity was determined by reference to Zhao et al. with slight modifications (Zhao et al., 2022). ANS was used as a fluorescence probe to measure the surface hydrophobicity. SPP hydrolysis product was dissolved in PBS (0.01 mol/L pH 7.0) to prepare different concentrations (0.1, 0.2, 0.3, 0.4 and 0.5 mg/mL). Take the prepared solution (4 mL) and 40 μL ANS (8.0 mmol/L, pH 7.0) mixed. After reacting in the dark for 60 min, the fluorescence spectrometer (F-4600 Hitachi Japan) was used to measure at 390 nm and 470 nm excitation wavelength and emission wavelength respectively, and the slit width was 5 nm. Linear relationship between fluorescence intensity and protein concentration. The slope corresponds to the surface hydrophobicity H_0_ of the protein.

### 2.10 X-ray diffractometer analysis (XRD)

XRD was performed by referencing the method of Hu et al. with slight modifications (Hu and Li, 2022). Analysis using an intelligent rotary target X-ray diffractometer [SmartLab (9 KW), Japan]. Put SPP hydrolysis product on the sample container, and scan at the rate of 10°C/min in the range of 10°C–50°C.

### 2.11 Differential scanning calorimetry analysis (DSC)

DSC was performed by referencing Hu et al. with slight modifications (Hu and Li, 2022). Analysis was performed using DSC (DSC25, TA Instruments Waters, United States). Put SPP hydrolysis product on the sample container, and scan it at a rate of 5°C/min within the range of 20°C–100°C.

### 2.12 Ultrafiltration fractionation of SPP hydrolysate

SPP hydrolysate (2 mg/mL) was filtered sequentially using an ultrafiltration unit (ULRC0300150P, Hangzhou Kebaite Filter Equipment Co., China) through two ultrafiltration membranes with molecular weight (MW) cut-off of 10 and 30 kDa, respectively. Three fractions with MWs<10 kDa, 10–30 kDa and >30 kDa were obtained. The concentration of SPP hydrolysate was measured by Folin phenol method, and then the peptide content of different grades was calculated according to Eq. 15.

$$Content\left(\%\right)=\frac{C\cdot V}{m}\times 100$$ (15)

Where, *C*: concentration of SPP hydrolysate (mg/mL); *V*: Volume of SPP hydrolysate (mL); *m*: Total weight of SPP hydrolysate 100 mg.

### 2.13 Atomic force microscopy analysis (AFM)

Atomic force microscopy is a reference to Quaisie et al. with slight modifications (Quaisie et al., 2021). AFM (Shimadzu spm-9700 ht, Japan) was used to analyze the surface morphology of SPP hydrolysis product. Take 0.001 g sample, disperse it in 10 mL ethanol, and then ultrasonic disperse it well. Take 10 mL, The sample is dried on the mica substrate under the infrared lamp for 20 min and then tested. Analytical conditions: scanning area 8.0 × 8.0 µm^2^, scanning frequency 1 Hz. AFM images were analyzed by Gwyddion software.

### 2.14 Measurement of antioxidant properties

#### 2.14.1 DPPH radical scavenging rate

DPPH radical scavenging rate was determined by referring to Martin, Ge and Wang et al. with slight modifications (Pyrzynska and Pękal, 2013; Ge et al., 2022; Wang et al., 2023). The SPP hydrolysis products (0.2, 0.4, 0.6, 0.8, 1 mg/mL) were dispersed in phosphate buffer (0.01 mol/L pH 7.0). First, 2 mL of the prepared sample was taken, followed by 2 mL of DPPH (0.04 mg/mL) solution, mixed well, incubated in a water bath at 25°C for 20 min, and then the absorbance value was measured at 517 nm. The DPPH radical scavenging rate was calculated according to Eq. 16.

$$DPPH\;radical\;scavenging\;rate\;\left(\%\right)=\left(\frac{A_{0}-A_{1}}{A_{0}}\right)\times 100$$ (16)

Where: *A*_0_: absorbance value of blank group; *A*_1_: absorbance value of sample group.

#### 2.14.2 Determination of Fe^2+^ chelation rate

Fe^2+^ chelation rate was determined by reference to the method of He and Ge et al. with slight modifications (He L. et al., 2021; Ge et al., 2022). SPP hydrolysis products (0.05, 0.1, 0.15, 0.2, 0.25 mg/mL) were dissolved in phosphate buffer (0.01 mol/L pH 7.0). Take 3 mL of the prepared solution, add 0.05 mL FeCl_2_ (2 mmol/L) and 0.1 mL philozine (5 mmol/L) successively, Fully mixed, incubate in a water bath at 25°C for 10 min, and then measure the absorbance value at 562 nm. The Fe^2+^ chelation rate was calculated according to Eq. (17).

$${Fe}^{2+}\;chelation\;rate\;\left(\%\right)=\left(\frac{A_{0}-A_{1}}{A_{0}}\right)\times 100$$ (17)

Where, the *A*_0_:absorbance value of the blank group and; the *A*_1_:absorbance value of the sample group.

#### 2.14.3 Determination of reducing power

Reducing power can effectively display the antioxidant activity of chemicals in food (Wu et al., 2015). Determination of reducing power was determined by referring to Ge et al. with slight modifications (Ge et al., 2022). The SPP hydrolysis product (0.5 mg/mL) was dissolved in phosphate buffer (0.01 mol/L pH 7.0). First, take 2 mL of the prepared solution. Add 2 mL of potassium K_2_Fe(CN_6_) (0.06 mol/L) solution, mix well, Incubate in a water bath at 50°C for 20 min. Add 2 mL of C_2_HCL_3_O_2_ (0.3 mol/L). Terminate the proceeding of the reaction. After centrifugation (10,000 r/min for 10 min), Take on 4 mL of supernatant, add 2.5 mL of distilled water and 0.5 mL FeCl_3_ (0.06 mol/L) solution, mix well, and stand still for 10 min. Measure the absorbance value at 700 nm, and use the absorbance value to express the reduction force.

### 2.15 Statistical analysis

All experiments were performed in three replicates and the results are expressed as mean ± standard deviation. P< 0.05 means statistically significant. Analysis was performed using SPSS 26.0 software and plotting was performed using Origin (2017) software.

## 3 Results and discussion

### 3.1 Effect of ultrasonic pretreatment with different frequencies on the hydrolysis of SPP

**FIGURE 1:**
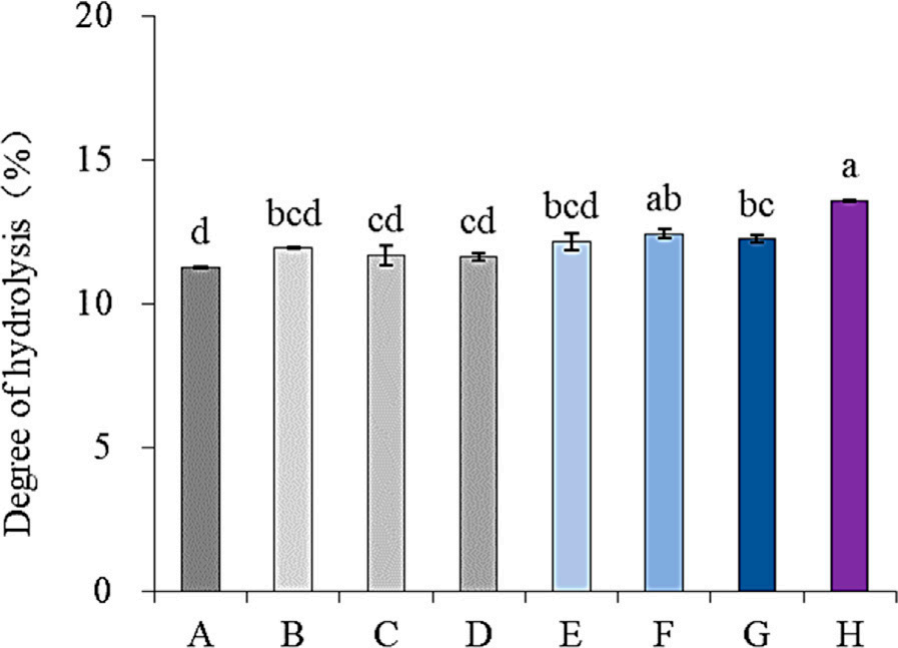
Effects of ultrasonic pretreatment with different frequencies on hydrolysis degree of SPP. **(A)**-traditional, **(B)**-22 kHz, **(C)**-28 kHz, **(D)**-40 kHz, **(E)**-2228 kHz, **(F)**-2240 kHz, **(G)**-2840 kHz and **(H)**-222840 kHz).

Figure 1 shows the effect of different ultrasonic frequencies on the degree of hydrolysis of SPP. Tri-frequency ultrasonic pretreatment remarkably enhanced the protein hydrolysis by protease enzyme (*p* < 0.05), indicating its significance in promoting proteolysis of SPP. Moreover, multi-frequency ultrasound can also increase cavitation effects (Hu et al., 2015). These findings are consistent with a previous report by Quaisie et al, (2021) documenting improved enzymatic hydrolysis of sea cucumber protein in response to ultrasonic pretreatment. Therefore, the tri-frequency ultrasound-assisted pretreatment method was selected for SPP.

### 3.2 Effect of traditional and tri-frequency ultrasound pretreatment on SPP hydrolysis under different conditions

**FIGURE 2:**
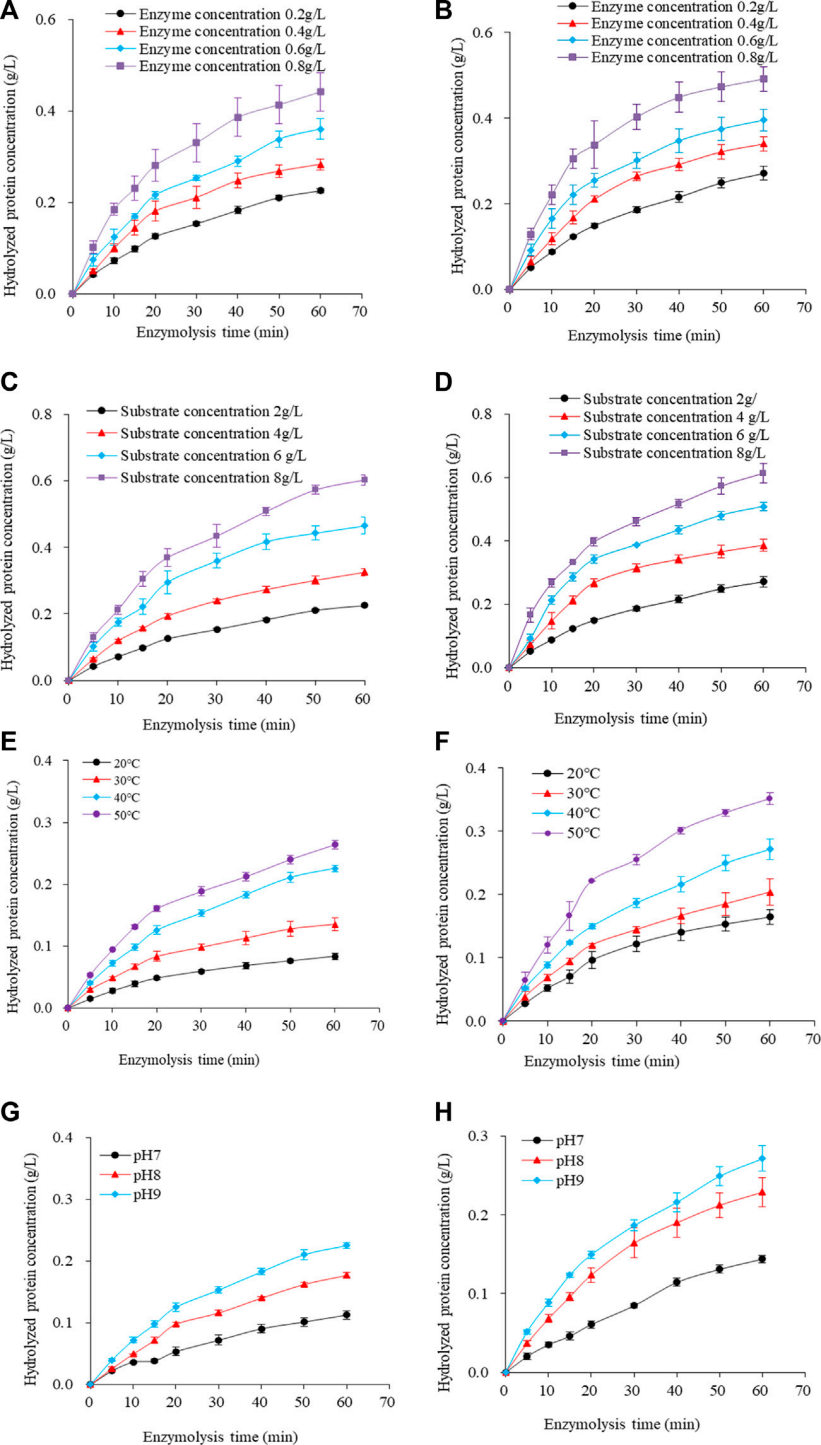
Hydrolysis diagram of SPP pretreated by **(A)** traditional and **(B)** tri-frequency ultrasound at different substrate concentration levels. (pH 9; temperature 40°C; enzyme concentration 0.2 g/L). Hydrolysis patterns of SPP pretreated with **(C)** traditional and **(D)** tri-frequency ultrasound at different enzyme concentration levels. (pH 9; temperature 40°C; substrate concentration 2 g/L). Hydrolysis patterns of SPP pretreated with **(E)** conventional and **(F)** tri-frequency ultrasound at different temperature levels. (pH 9.0; substrate concentration 2 g/L; enzyme concentration 0.2 g/L). Hydrolysis diagram of SPP with **(G)** traditional and **(H)** tri-frequency ultrasonic pretreatment at different pH levels. (temperature 40°C; substrate concentration 2 g/L; enzyme concentration 0.2 g/L).

Figure 2 shows the changes in enzyme hydrolysis of traditional and ultrasonication-treated SPP under different enzyme and substrate concentrations, temperatures, and pH. The typical change curve of protein enzyme hydrolysis is presented. The amount of hydrolyzed protein increased continuously during enzymatic proteolysis as a function of time, with a faster reaction rate of up to 20 min and a gradual increase afterward. These results are in agreement with Dabbour et al. (2020) reporting enzyme hydrolysis of sunflower proteins.

The SPP hydrolysate concentration was enhanced with increasing enzyme concentration during hydrolysis at 40°C, pH 9.0, and substrate concentration of 2 g/L (Figures 2A,B), indicating a positive correlation between the degree of hydrolysis and the enzyme concentration within a specific range. For example, the amount of ultrasound-pretreated hydrolyzed protein increased by 11.31% (0.492 ± 0.029 g/L) compared to traditional hydrolysate (0.442 ± 0.042 g/L) at an enzyme concentration of 0.8 g/L (*p* < 0.05). Likewise, an increase in SPP hydrolysate concentration was achieved as the substrate concentration was raised in the hydrolysis reaction performed using 0.2 g/L enzyme at pH 9.0°C and 40°C (Figures 2C,D). This also reveals a positive association between the degree of hydrolysis and substrate concentration up to a certain range. Compared with the traditional untreated hydrolyzed protein concentration (0.602 ± 0.015 g/L), an overall enhancement of 1.827% (0.613 ± 0.013 g/L) was observed for the ultrasonically pre-treated hydrolysate at a substrate concentration of 8 g/L (*p* < 0.05).

A significant increase in SPP hydrolysate was observed after enzymatic reaction at different temperatures (Figures 2E,F) and pH 9.0 using enzyme and substrate concentrations of 0.02 g/L and 2.0 g/L, respectively. At an optimum temperature of 50°C, 0.351 ± 0.009 g/L of hydrolyzed protein was obtained following ultrasonic pretreatment, with an overall increase of 32.955% compared to the traditional hydrolysate (0.264 ± 0.007 g/L) (*p* < 0.05). Reaction pH had a similar effect on the hydrolysis of SPP, achieving 0.272 ± 0.016 g/L of hydrolysate after ultrasound pretreatment at pH 9.0°C and 40°C, using enzyme and substrate concentrations of 0.2 g/L and 2.0 g/L, respectively (Figures 2G,H). This indicates a 20.88% increase in hydrolysate concentration compared to traditional hydrolysate (0.225 ± 0.005 g/L) at similar conditions (*p* < 0.05).

The reason for a higher degree of hydrolysis after pretreatment with tri-frequency ultrasonic waves than the traditional under the same conditions of enzymatic digestion could be attributed to the cavitation effect of ultrasound (Abdualrahman et al., 2017), mechanical action (Wang et al., 2015), and Van der Waals forces (Zhang et al., 2015), working together to ensure the breakdown of covalent bonds within protein molecules (Umego et al., 2021), resulting in protein unfolding and fragmentation. Ultrasound can greatly enhance the biological activity of hydrolysates, thereby accelerating the speed of protein hydrolysis and improving the efficiency of protein hydrolysis. Furthermore, the altered protein structure exposed the protein molecule fragments to become readily accessible to the enzyme, leading to enhanced degradation. Therefore, tri-frequency ultrasound treatment can accelerate the enzyme hydrolysis efficiency of SPP.

### 3.3 Effect of tri-frequency ultrasound pretreatment on kinetic parameters (k_m_ and k_A_) and initial reaction rate

**FIGURE 3:**
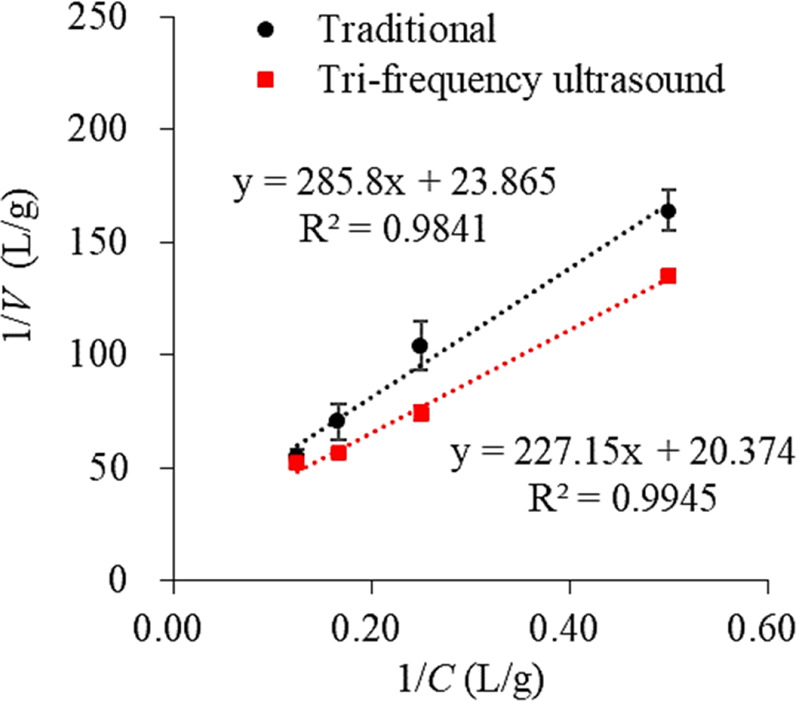
The reciprocal graph of the reciprocal of the initial reaction rate (1/V) and substrate concentration (1/C) of the enzymatic hydrolysis of SPP by traditional and tri-frequency ultrasound pretreatment.

The reaction rate catalyzed by alkaline proteases generally follows the Michaelis-Menten kinetic model (Dabbour et al., 2018). The Mie’s constant (*k*_m_) is fundamental in the kinetics of the hydrolysis reaction, which is inversely proportional to the reaction rate; the lower the *k*_m_ value, the faster the reaction rate. The effect of sonication on the kinetic parameters (*k*_m_ and *k*_A_) was determined based on an inverse plot of the initial reaction rate (1/*V*) versus initial protein concentration (1/*C*), which was linear in the middle, with linear regression coefficient R values of 0.9841 and 0.9945, respectively (Figure 3). Table 1 shows the kinetic parameters and initial reaction rates for traditional and sonication pretreated hydrolysis reactions. After sonication, the *k*m value decreased by 6.369% while k_A_ increased by 16.746%. This is consistent with the study of Jin et al. employing sonication pretreatment for the enzymatic hydrolysis of maize gluten protein, where a 26.1% decrease in the *k*_m_ value and 7.3% enhancement in the *k*_A_ was observed compared to traditional hydrolysate (Jin et al., 2015). Furthermore, the initial reaction rates for ultrasound-pretreated hydrolysis increased with the increasing protein concentrations, showing 21.311%, 40.625%, 23.239%, and 4.918% increments at substrate concentrations of 2.0 g/L, 4.0 g/L, 6.0 g/L, and 8.0 g/L, respectively (Table 1). These results demonstrate that tri-frequency ultrasonic pretreatment significantly enhances the enzymatic hydrolysis of SPP by increasing the initial reaction rate.

**TABLE 1:** Effect of traditional and tri-frequency ultrasound pretreatment on kinetic factors and initial reaction rates.

Method of enzymolysis	*k*_m_/*k*_A_ *E*_c_ (min)	1/*k*_A_ *E*_c_ (L· min/g)	*k*_m_ (g/L)	*k*_A_ (1/min)	Initial reaction rate (g/L· min)			
					2 (g/L)	4 (g/L)	6 (g/L)	8 (g/L)
Traditional	284.123 ± 7.914	25.004 ± 5.633	11.835 ± 2.100	0.209 ± 0.042	0.0061 ± 0.0003^b^	0.0096 ± 0.0009^b^	0.0142 ± 0.0016^b^	0.0183 ± 0.0012^b^
Tri-frequency ultrasound	227.050 ± 7.403	20.510 ± 0.333	11.078 ± 0.525	0.244 ± 0.004	0.0074 ± 0.0002^a^	0.0135 ± 0.0006^a^	0.0175 ± 0.0002^a^	0.0192 ± 0.0003^a^
Increment (%)			−6.396	16.746	21.311	40.625	23.239	4.918

Mean ± Standard deviation of triplicate determinations.

Lowercase letters indicate significant difference between groups.

### 3.4 Effect of tri-frequency ultrasound pretreatment on the rate constant (k) of the enzyme hydrolysis reaction

**FIGURE 4:**
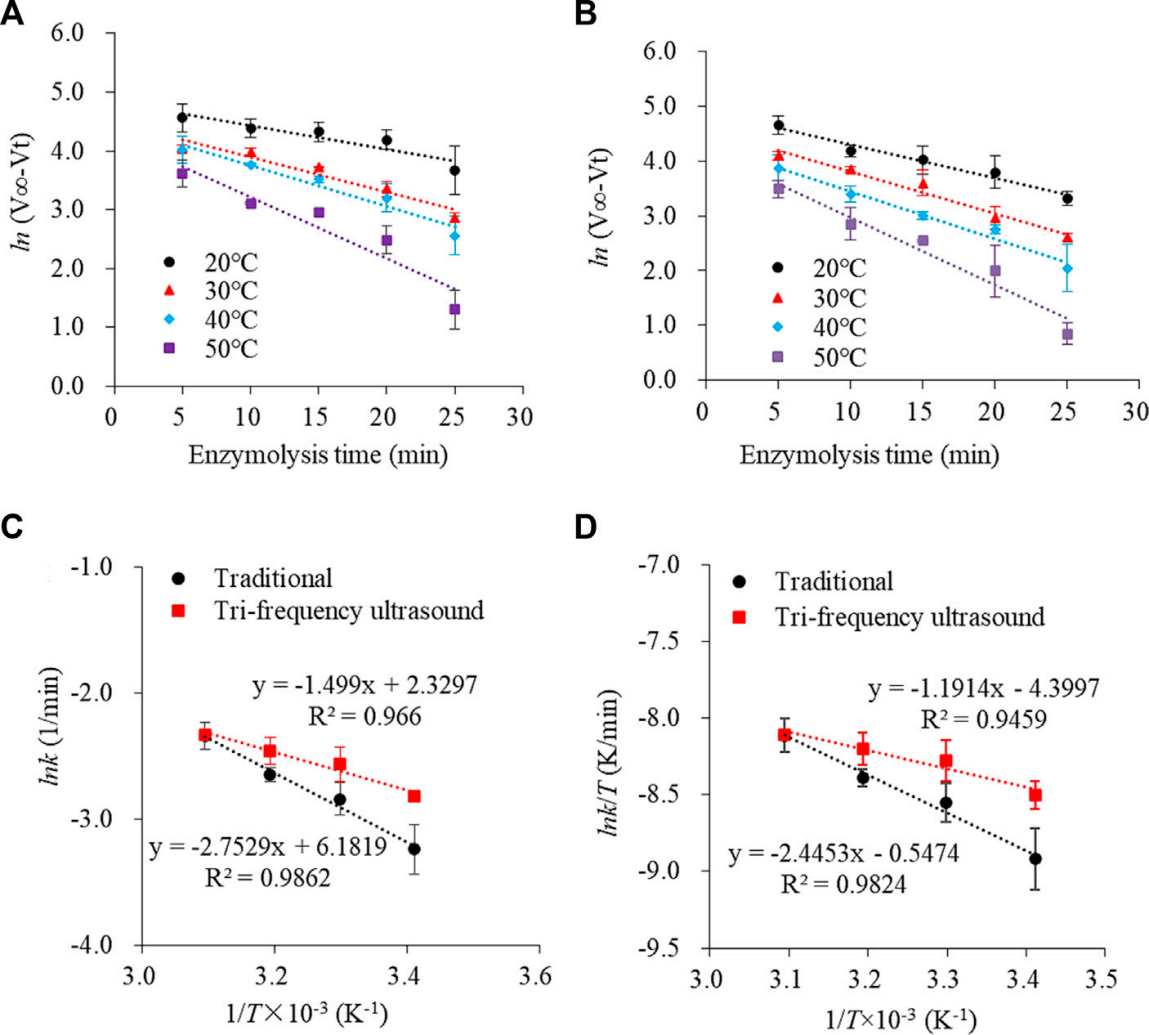
Plot of ln (V_∞_- V_t_) versus enzymolysis time (min) at different temperatures for traditional and tri-frequency ultrasound pretreatment (substrate concentration 2 g/L; enzyme concentration 0.2 g/L; pH 9). Traditional and tri-frequency ultrasound pretreatment of linear fitting curve for enzymolysis of SPP, **(C)** Ink and **(D)** Ink. T^-1^ against 1/T × 10^–3^.

The rate constant (*k*) is important to quantify changes in the kinetics of enzyme hydrolysis, directly reflecting the speed of the reaction rate. It is usually related to the reaction temperature, medium, and enzyme. The alterations in the spatial structure of SPP after ultrasonication would inevitably alter the rate constant. The curves depicting SPP (traditional and ultrasound pre-treated) hydrolysis ln (V_∞_-V_t_) versus reaction time (*t*) showed linear regression correlation coefficients above 0.9 (Figures 4A,B), which was consistent with the primary reaction kinetic model (Ayim et al., 2018).

The rate constant (*k*) increased gradually with a rise in the reaction temperature of the enzyme hydrolysis process (Table 2), which could be attributed to the enhanced irregular motion of substrate and enzyme molecules in the reaction system, increasing their collision frequency and in turn, the reaction rate (Musa et al., 2019), because ultrasonication could induce structural changes in SPP, exposing more active centers to escalate the enzyme-catalyzed reaction. Thus, tri-frequency ultrasound pretreatment can improve the rate of enzyme-catalyzed proteolysis of SPP at higher temperatures.

**TABLE 2:** Reaction rate constant (1/min) and *R* ^2^ for traditional and tri-frequency ultrasound pretreated enzymolysis at various absolute temperatures.

Temperature (°C)	Treatment			*R* ^2^	
	Traditional	Tri-frequency ultrasound			
	*k*_c_ (1/min)	*k* (1/min)	*ku* (1/min)	Traditional	Tri-frequency ultrasound
20	0.0400 ± 0.0074^b^	0.0613 ± 0.0071^a^	0.0213 ± 0.0134	0.8592	0.9658
30	0.0591 ± 0.0071^b^	0.0765 ± 0.0098^a^	0.0174 ± 0.0031	0.9258	0.969
40	0.0712 ± 0.0041^b^	0.0864 ± 0.0092^a^	0.0152 ± 0.0057	0.9498	0.9779
50	0.0974 ± 0.0107^b^	0.1245 ± 0.0017^a^	0.0272 ± 0.0099	0.8979	0.9498

Mean ± Standard deviation of triplicate determinations.

Lowercase letters indicate significant difference between groups.

### 3.5 Effect of tri-frequency ultrasound pretreatment on thermodynamic parameters of the hydrolysis reaction

To elucidate the mechanism of enzymatic hydrolysis of SPP, thermodynamic parameters, such as *E*_a_, *ΔH*, *ΔS and ΔG* were determined. *E*_a_ represents the minimum energy required to convert a molecule from the normal steady state to the reaction state (Ren et al., 2018) and generally falls in the range of 40–400 kJ/mol for a thermodynamically feasible reaction; *E*_a_ < 40 kJ/mol signifies a faster reaction rate, while *E*_a_ > 400 kJ/mol indicates non-feasibility. The Arrhenius plot of ln *k* versus 1/*T* illustrated that *E*_a_ for both traditional and ultrasonically pretreated hydrolysis reaction was lower than 40 kJ/mol, implying that the reaction proceeded faster (Figures 4C,D). Moreover, the *E*_a_ of pupa protein hydrolysis after ultrasound treatment decreased by 21.943% (*p* < 0.05) compared to traditional hydrolysate (Table 3); the larger the *E*_a_, the slower the reaction. Similarly, the *ΔH* for the proteolysis reaction after ultrasonication was reduced by 24.712% compared to traditional hydrolysate. The activation entropy *ΔS* of a reaction is usually negative, indicating the formation of activation complexes as a process of increasing order (Qu et al., 2013; Wu et al., 2018). For the proteolytic reactions involving SPP, *ΔH* was positive while *ΔS* was negative. *ΔG* depends on the combined changes in *ΔS* and *ΔH.* Accordingly, a decrease in *ΔH* and an increase in *ΔS* resulted in decreased *ΔG*. At 50°C, *ΔG* for the ultrasonically treated pupa protein hydrolysis reaction was reduced by 4.544% compared to traditional hydrolysate, thus requiring less energy for the enzymatic hydrolysis.

**TABLE 3:** Thermodynamic factors for traditional and tri-frequency ultrasound pretreated enzymolysis.

Treatment	*E*_a_ (kJ/moL)	*∆H* (kJ/moL)	*∆S* (J/moL^·^k)	*∆G*			
				20 °C	30 °C	40 °C	50 °C
Traditional	22.887 ± 7.182^a^	20.330 ± 7.182^a^	−170.235 ± 3.461^a^	70.405 ± 6.187^a^	72.108 ± 6.154^a^	73.810 ± 6.120^a^	75.512 ± 6.086^a^
Tri-frequency ultrasound	17.865 ± 0.594^b^	15.306 ± 0.593^b^	−175.148 ± 1.418^b^	66.826 ± 1.005^b^	68.579 ± 1.019^b^	70.329 ± 1.033^b^	72.081 ± 1.047^b^
Increment (%)	−21.943	−24.712	−2.886	−5.083	−4.900	−2.467	−4.544

Mean ± Standard deviation of triplicate determinations.

Lowercase letters indicate significant difference between groups.

### 3.6 Analysis of the second order rate extraction model

**FIGURE 5:**
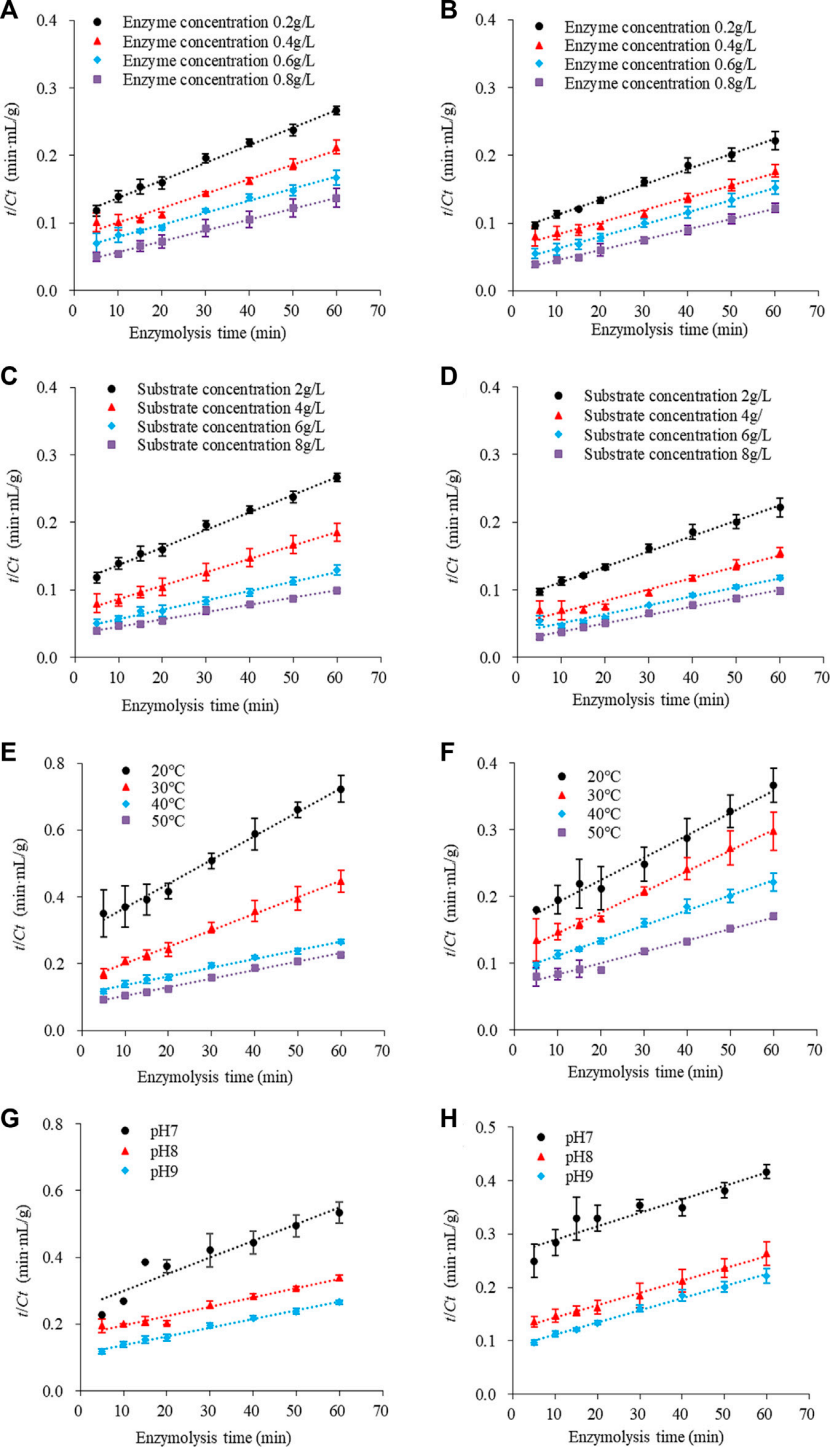
Second order model: kinetics of enzymolysis of SPP in **(A)** traditional and **(B)** tri-frequency ultrasound pretreatment at different substrate concentrations. Enzymolysis kinetics of SPP in **(C)** traditional and **(D)** tri-frequency ultrasound pretreatment groups at different enzyme concentrations. Kinetics of enzymolysis of SPP in **(E)** traditional and **(F)** tri-frequency ultrasound groups at different temperatures. Kinetics of enzymolysis of SPP of SPP in **(G)** traditional and **(H)** tri-frequency ultrasound pretreatment groups at different pH values.

The second-order rate kinetics of SPP hydrolysis are shown in Figure 5, along with the deduced kinetics parameters *C*_S_ and *K* (Table 4; Table 5; Table 6; Table 7) under different reaction conditions. The reaction parameters (enzyme and substrate concentrations, temperature, and pH) were directly proportional to the hydrolysate concentration over a range. A greater *C*s value indicated higher amounts of protein hydrolysate in the reaction. This is in line with the findings of Agu et al. (2022) where the extraction of *Glycyrrhiza glabra* seed oil was proportional to temperature increase. The correlation coefficients of the experimental models were above 0.85, The correlation coefficient is mostly as high as 0.99. Therefore, the second-order model appears suitable for analyzing the kinetics of enzymatic proteolysis processes.

**TABLE 4:** Second-order model: under different enzyme concentration, the enzymolysis kinetics of SPP was pretreated with traditional and three-frequency ultrasound.

Treatment	Model parameters	Enzyme concentration (g/L)			
		0.2	0.4	0.6	0.8
Traditional	*C*_*S*_	45.005	63.452	80.128	122.249
	*K*	0.190	0.113	0.087	0.042
	R_Adj_^2^	0.9927	0.978	0.9912	0.9973
Tri-frequency ultrasound	*C*_*S*_	56.306	77.042	114.416	170.068
	*K*	0.137	0.094	0.042	0.023
	R_Adj_^2^	0.9943	0.9857	0.9981	0.9975

**TABLE 5:** Second-order model: under different Substrate concentration, the enzymatic hydrolysis kinetics of SPP was pretreated with traditional and three-frequency ultrasound.

Treatment	Model parameters	Substrate concentration (g/L)			
		2	4	6	8
Traditional	*C*_*S*_	45.005	74.850	115.741	144.93
	*K*	0.190	0.089	0.053	0.043
	R_Adj_^2^	0.9929	0.9977	0.9925	0.9944
Tri-frequency ultrasound	*C*_*S*_	56.306	98.814	132.625	190.050
	*K*	0.137	0.060	0.0437	0.023
	R_Adj_^2^	0.9943	0.963	0.9615	0.9963

**TABLE 6:** Second-order model: the kinetics of enzymatic hydrolysis of SPP under different temperatures by traditional and three-frequency ultrasonic pretreatment.

Treatment	Model parameters	Temperature (°C)			
		20	30	40	50
Traditional	*C*_*S*_	16.898	32.960	45.005	62.893
	*K*	0.486	0.184	0.190	0.101
	R_Adj_^2^	0.9925	0.9958	0.9927	0.9922
Tri-frequency ultrasound	*C*_*S*_	31.506	43.592	56.306	75.872
	*K*	0.305	0.170	0.137	0.102
	R_Adj_^2^	0.9839	0.9939	0.9943	0.9826

**TABLE 7:** Second-order model: under different pH, the enzymatic hydrolysis kinetics of SPP was pretreated with traditional and three-frequency ultrasound.

Treatment	Model parameters	pH		
		7	8	9
Traditional	*C*_*S*_	19.952	29.377	45.005
	*K*	0.502	0.414	0.190
	R_Adj_^2^	0.8914	0.9705	0.9927
Tri-frequency ultrasound	*C*_*S*_	18.882	41.288	56.306
	*K*	1.122	0.255	0.137
	R_Adj_^2^	0.8847	0.9939	0.9943

### 3.7 Surface hydrophobicity analysis

**FIGURE 6:**
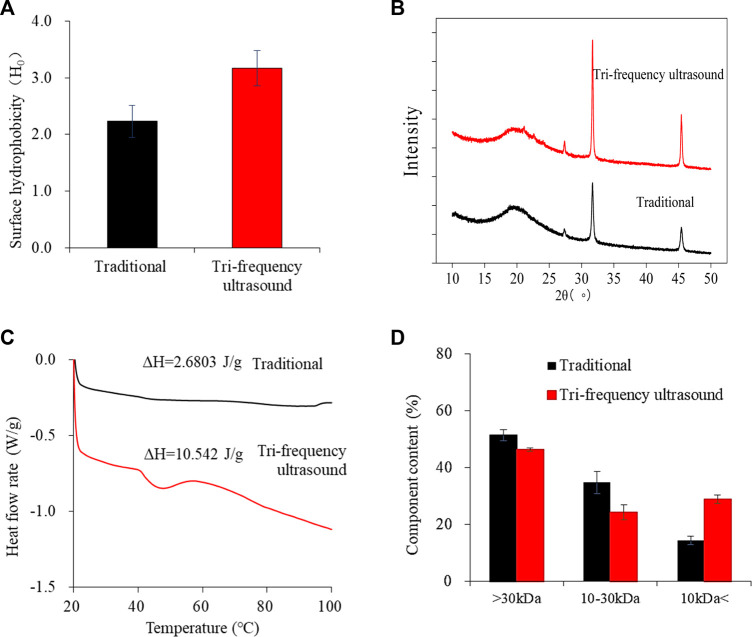
Effects of traditional and tri-frequency ultrasound treatment of SPP hydrolysate. **(A)** Surface hydrophobicity (H_0_) **(B)** XRD **(C)** DSC **(D)** Ultrafiltration classification.

The hydrophobicity of the ultrasonically pretreated SPP hydrolysate was higher than that of traditional hydrolysate (Figure 6A) (*p* < 0.05). The increase in surface hydrophobicity may be because of the expansion of proteins by ultrasonic waves, exposing the hydrophobic regions (Zhang J. et al., 2022). In a similar report, the surface hydrophobicity of whey protein was enhanced with prior ultrasonic treatment (300 W, 15 min) (Wu et al., 2018). Therefore, tri-frequency ultrasound has an enhanced effect on the H0 of SPP enzymatic hydrolysis products.

### 3.8 XRD analysis

XRD method can analyze alterations in the protein crystal structure after ultrasound treatment (Wang et al., 2023). Figure 6B shows the XRD spectra of the hydrolysates of SPP pretreated traditionally and by tri-frequency ultrasound. A broad peak was observed at 2θ = 20, indicating the amorphous structure of SPP (Yuan et al., 2021). Additionally, multiple sharp characteristic peaks observed at 2θ = 27.330, 31.700 and 45.420 suggested the presence of crystalline regions. The intensity of these peaks increased for ultrasound-pretreated hydrolysate, reflecting an enhancement in crystallinity. (Hu and Li, 2022). These results are consistent with a previous report by Hu and Li, (2022) showing a rise in the peak intensity of soy protein isolate nanofibrils with a simultaneous increase in the amplitude of ultrasonic waves pretreatment (amplitude of 20%, 60%, and 80%). In conclusion, tri-frequency ultrasound pretreatment enhanced the crystalline regions within the hydrolysates of SPP.

### 3.9 DSC analysis

In a DSC curve, the maximum peak temperature indicates the transformation temperature during protein denaturation, and the denaturation temperature can reflect the thermal stability and aggregation of protein molecules (Hu and Li, 2022). The DSC plots of SPP hydrolysate before and after ultrasound treatment subjected to heating from 20°C to 100°C showed a broad heat absorption peak at 45°C (Figure 6C). A similar broad heat absorption peak at approximately 82°C was observed in lupin proteins before and after ultrasound treatment (Lo et al., 2022). This typical peak indicates hydrogen bonding and electrostatic interactions within the protein, which determines the denaturation point (Lo et al., 2022). Compared with the traditional, the denaturation temperature of ultrasonically pretreated hydrolysate shifted from 44.913°C ± 0.434°C to 46.800°C ± 0.000°C, and the enthalpy of thermal change *ΔH* increased significantly from 2.6803 ± 0.218 J/g to 10.542 ± 0.090 J/g (*p* < 0.05). Ultrasound treatment could probably affect the hydrogen bonding between protein molecules (Zhang W. et al., 2022), requiring more heat absorption for complete protein denaturation and enhancing thermal stability.

### 3.10 Fractionation of SPP hydrolysate by ultrafiltration

The ultrafiltration classification diagram of SPP hydrolysate illustrates a reduction in molecular weight after ultrasound pretreatment (Figure 6D). This could be because of the degradation of protein molecules into smaller peptides by the combined action of tri-frequency ultrasonic waves and enzymolysis, significantly changing the molecular weight of SPP hydrolysate (*p* < 0.05).

### 3.11 Atomic force microscopy analysis

**FIGURE 7:**
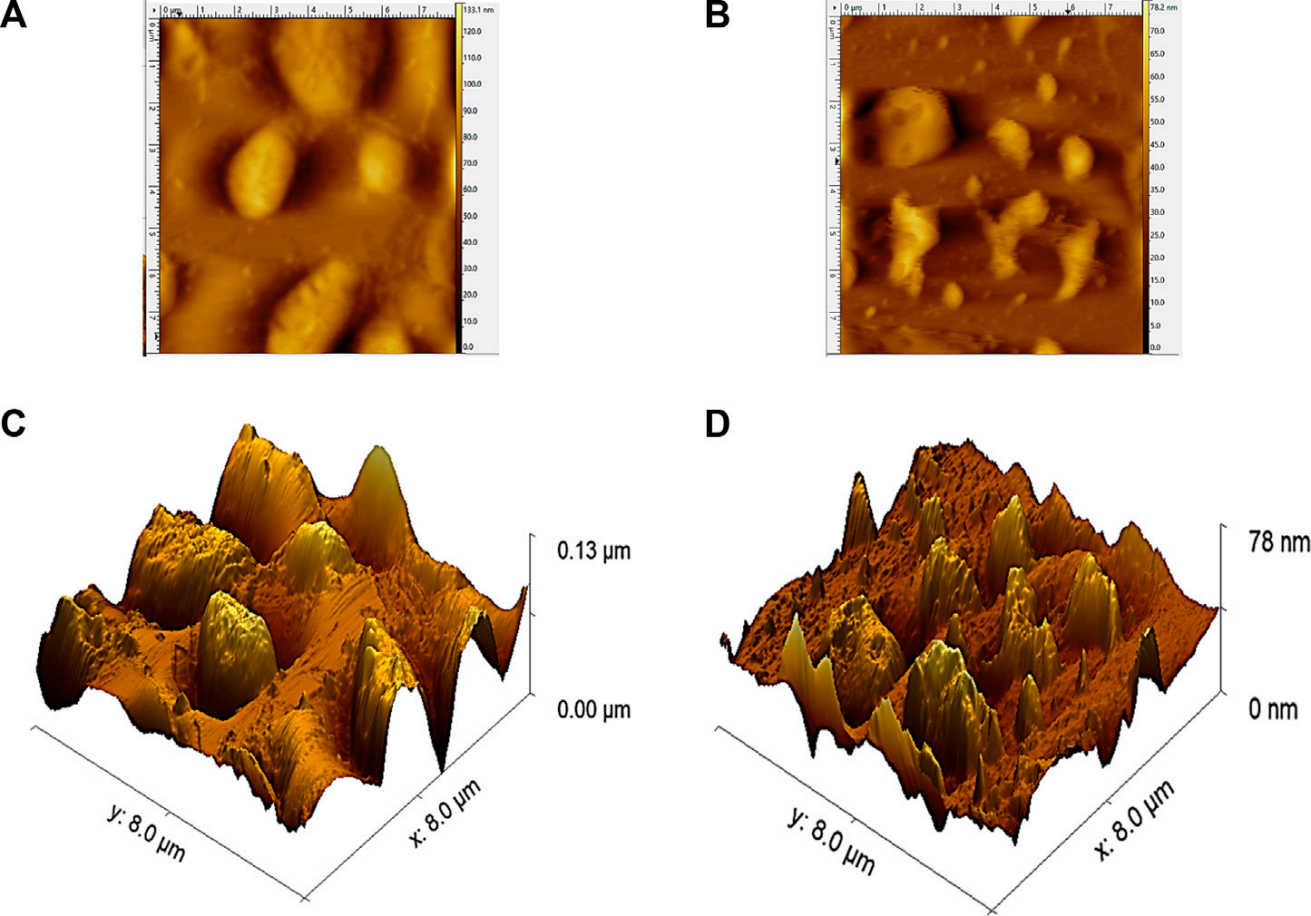
Effects of traditional and tri-frequency ultrasound treatment on the AFM morphology of SPP hydrolysate. **(A)** Traditional two-dimensional structure **(B)** tri-frequency ultrasound two-dimensional structure **(C)** Traditional tri-dimensional structure **(D)** tri frequency ultrasound tri-dimensional structure.

The surface morphology of SPP hydrolysate, treated conventionally and by ultrasonic waves, was analyzed by atomic force microscopy (AFM) (Figure 7). Hydrolysate obtained after conventional treatment showed a compact structure. In contrast, larger aggregates broke into smaller irregular fragments after ultrasonic action, which appeared loose and uniform in size (Jin et al., 2021). In addition, the surface roughness of the hydrolysate increased after ultrasonic treatment; the rougher the protein surface, the more convenient the enzyme and protein interaction (Xu et al., 2020). In a similar study, feather protein particles became porous after ultrasound treatment, conducive for the smooth entry of enzymes into protein molecules to trigger enzymatic hydrolysis (Chen et al., 2017; Hong et al., 2022). The results demonstrate modifications in the surface morphology and nanostructure of SPP hydrolysate after ultrasonic pretreatment.

### 3.12 Antioxidant activity of the SPP hydrolysate

The antioxidant activity of SPP hydrolysate, both traditional and ultrasound pretreated, was evaluated (Table 8). The IC_50_ of DPPH and Fe^2+^ chelation rates in the traditional group were 4.725 ± 0.091 mg/mL and 4.510 ± 0.193 mg/mL, respectively, while the same values decreased to 12.8% (4.119 ± 0.078 mg/mL) and 9.2% (4.095 ± 0.244 mg/mL) after ultrasonic treatment (*p* < 0.05). Moreover, at a concentration of 0.5 mg/mL, the reducing power of the ultrasonically treated sample increased by 8.800% (1.043 ± 0.008 mg/mL) compared to that of the traditional (0.975 ± 0.029 mg/mL) (*p* < 0.05). Sonication-driven exposure of peptide chains of aromatic amino acid residues with high antioxidant capacity led to the enhancement in antioxidant properties of SPP hydrolysate (Bian et al., 2022). In a previous report by Yang et al. ultrasonic-assisted dual enzyme stepwise hydrolysis of low molecular weight peptides from bovine bone exhibited the highest antioxidant activity (*p* < 0.05) (Yang et al., 2021). Based on the results, the antioxidant effect of the SPP hydrolysate was considerably enhanced in response to tri-frequency ultrasound treatment.

**TABLE 8:** The effect of traditional and tri-frequency ultrasound on the antioxidant activity of SPP hydrolysate.

Treatment	IC_50_ of DPPH radical scavenging (mg/mL)	IC_50_ of Fe^2+^ chelation (mg/mL)	Reducing power
Traditional	4.725 ± 0.091^a^	4.510 ± 0.193^a^	0.500 ± 0.029^a^
Tri-frequency ultrasound	4.119 ± 0.078^b^	4.095 ± 0.244^b^	0.975 ± 0.008^b^
Increment (%)	−12.825	−9.201	8.800

Mean ± Standard deviation of triplicate determinations.

Lowercase letters indicate significant difference between groups.

## 4 Conclusion

In this study, the effects of tri-frequency ultrasound pretreatment on the thermodynamic and kinetics parameters of SPP hydrolysate was investigated, with noticeable improvement in the hydrolysis efficiency post ultrasonic action under different substrate and enzyme concentrations, temperatures, and pH conditions. Furthermore, ultrasonic pretreatment led to decreased *k*_m_ and increased *k*_A_ and rate constant (*k*) during kinetic studies, indicating an improved affinity between substrate and enzyme. Thermodynamic analysis showed a reduction in *E*_a_ and *ΔG* of the hydrolytic reaction after sonication treatment for a faster and less energy-requiring process. Moreover, the sonicated hydrolysate exhibited better thermal stability and crystallinity than the traditional because of the enhanced exposure of surface hydrophobic groups. The results indicate that the hydrolysis of silkworm pupa protein pretreated with three frequency ultrasound can produce peptides with high antioxidant activity, which has potential application prospects as natural antioxidants in the food industry. The current work contributes to more specific research on the separation, purification, and identification of antioxidant peptides from silkworm pupa proteins.

## Data Availability

The original contributions presented in the study are included in the article/supplementary material, further inquiries can be directed to the corresponding authors.

## Abbreviations

SPP, Silkworm pupa protein; ACE, Angiotensin-converting enzyme; *k*_m_, Mie’s constant; *k*_A_, Apparent decomposition rate constant; *k*, Reaction rate constant; Ea, Activation energy; ΔS, Entropy change; ΔH, Enthalpy change; ΔG, Gibbs free energy change; DSC, Differential scanning calorimetry; XRD, X-ray diffraction; AFM, atomic force microscopy.
